# Bioinformatics frameworks for single-cell long-read sequencing: unlocking isoform-level resolution

**DOI:** 10.1093/bib/bbaf655

**Published:** 2025-12-11

**Authors:** Saloni Bhatia, Matt A Field, Lionel Hebbard, Ulf Schmitz

**Affiliations:** Computational Biomedicine Lab, College of Science and Engineering, James Cook University, 1 James Cook Drive, Townsville, QLD 4811, Australia; Centre for Tropical Bioinformatics and Molecular Biology, James Cook University, 14-88 McGregor Road, Smithfield, QLD 4878, Australia; Centre for Tropical Bioinformatics and Molecular Biology, James Cook University, 14-88 McGregor Road, Smithfield, QLD 4878, Australia; Immunogenomics Lab, Garvan Institute of Medical Research, 384 Victoria St, Darlinghurst, NSW 2010, Australia; Menzies School of Health Research, Charles Darwin University, Red 9, Casuarina campus, Univ Drive North, Casuarina, NT 0811, Australia; Centre for Tropical Bioinformatics and Molecular Biology, James Cook University, 14-88 McGregor Road, Smithfield, QLD 4878, Australia; Storr Liver Centre, Westmead Institute for Medical Research, Westmead Hospital and University of Sydney, 176 Hawkesbury Rd, Westmead, Westmead, NSW 2145, Australia; Australian Institute for Tropical Health and Medicine, 1 James Cook Drive, Townsville, QLD 4811, Australia; Computational Biomedicine Lab, College of Science and Engineering, James Cook University, 1 James Cook Drive, Townsville, QLD 4811, Australia; Centre for Tropical Bioinformatics and Molecular Biology, James Cook University, 14-88 McGregor Road, Smithfield, QLD 4878, Australia; Centenary Institute, The University of Sydney, Building 93, Royal Prince Alfred Hospital Missenden Rd, Camperdown, NSW 2006, Australia

**Keywords:** alternative splicing, single-cell long-read sequencing, isoform quantification, differential isoform expression

## Abstract

Alternative splicing (AS) plays a key role in regulating gene expression, and its dysregulation is implicated in numerous human diseases, including cancer. While bulk RNA sequencing has advanced our understanding of AS, it cannot capture cellular heterogeneity or reliably reconstruct full-length isoforms, both of which underpin disease mechanisms and therapeutic responses. Single-cell RNA sequencing (scRNA-seq) is an established and a powerful approach to examine AS landscapes at single-cell resolution, enabling the identification of cell-specific aberrant splicing events that may contribute to disease. However, conventional scRNA-seq is limited by short read lengths, often preventing an accurate reconstruction of full-length transcript isoforms. This limitation is addressed by long-read RNA-seq (lrRNA-seq), which can sequence full-length RNA molecules, some exceeding 100 000 nucleotides in length. Thereby, lrRNA-seq enables more accurate characterization of isoform diversity, identification of novel splice variants, quantification of percent spliced-in values, and detection of fusion transcripts. The convergence of single-cell resolution and third-generation sequencing technologies has led to the development of single-cell long-read sequencing (SCLR-seq), a powerful approach that addresses the key constraints of bulk short-read RNA-Seq by providing isoform-level resolution and cell-type specificity. This review explores the growing utility of SCLR-seq, highlighting recent developments in bioinformatics tools and pipelines designed for SCLR-seq data analysis. We discuss how this emerging technology is transforming our understanding of isoform regulation and aberrant splicing in human diseases, and its potential to uncover novel diagnostic and therapeutic targets.

## Graphical Abstract

**Graphical Abstract:**
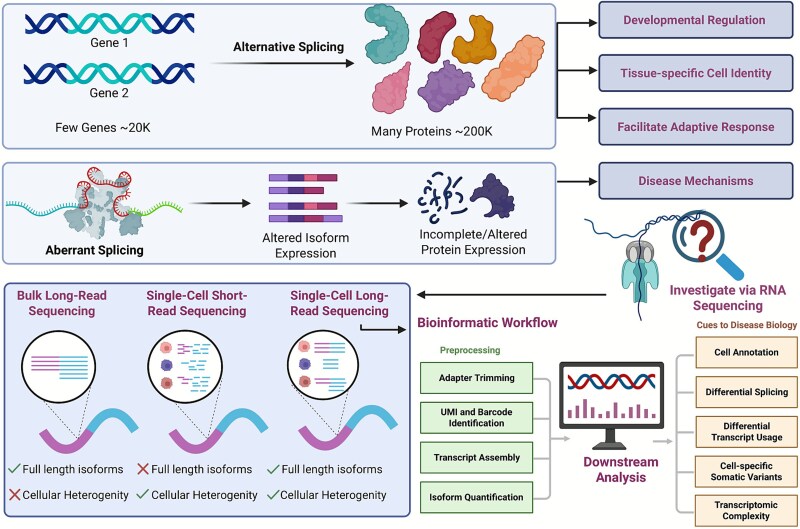

## Introduction

Important biological processes such as cell development and differentiation are directed by gene expression networks, often modulated by alternative splicing (AS) with over 30% of tissue-dependent transcript variants constituted by local splicing events [1]. In this context, alternative isoforms can be generated from the same gene by either differential processing of the pre-mRNA or by using alternative transcription start/end sites. A set of carefully balanced regulatory mechanisms interact with each other to tightly regulate AS spatiotemporally [2], meaning that any disruption in AS can lead to or mediate diseases. Numerous human hereditary diseases and cancers are associated with aberrant AS [3, 4].

### Profiling AS and isoform expression via RNA sequencing

To understand the role of AS in human diseases, it is important to accurately map and quantify AS events and decipher their functional significance. High-throughput RNA-Seq of patient-derived tissue samples has been useful for generating gene expression profiles that could be mined, e.g. for diagnostic and prognostic markers for clinical management. Such transcriptome profiling studies have largely relied on bulk short-read RNA sequencing (RNA-Seq) [5]; however, this approach fails to capture the genomic heterogeneity between different cell types within the same tissue. In fact, cellular heterogeneity has a significant impact on clinical outcomes altering the efficacy of some therapies [6]. To capture this heterogeneity, single-cell RNA-seq (scRNA-seq) is used to examine transcriptomic cell-to-cell variation. While bulk RNA-seq data are dominated by abundant cell types relative to rare cell types, scRNA-seq can capture information from both abundant and relatively rare cell types.

Beyond cellular heterogeneity and rare cell types, single-cell transcriptomics provides insights into cellular architecture and dynamic transcriptomic changes across time or experimental conditions. Every tissue contains functionally and morphologically diverse populations of cells in different states and spatial locations. Cell architecture in disease states has been investigated very well in tumors, e.g. many biological processes including development, immune responses, and cancer evolution are driven by cell-specific transcriptomic programs. Single-cell resolution is therefore critical to capture this diversity. For instance, rare cell types may express unique isoforms that are undetectable in bulk, and clonal populations within tumors may show distinct splicing patterns or mutational profiles.

However, transcript-level analysis in droplet-based scRNA-seq platforms such as 10× Genomics is inherently limited by protocol-induced biases. Depending on the chemistry employed (3′ or 5′ capture), these protocols selectively enrich for one end of the transcript, resulting in biased coverage concentrated at the 3′ or 5′ end and incomplete representation of the full transcript body. This results in uneven coverage across transcripts, often missing critical splice junctions or transcript start/end sites required for accurate isoform resolution.

Additionally, one end of each read is typically reserved for sequencing the cell barcode (CB) and unique molecular identifier (UMI), leaving only the remaining portion of the read, often a single-end read of ~90–100 bp, for transcript alignment. This reduced effective read length limits the ability to uniquely map reads, especially in repetitive or homologous regions, thereby inflating multi-mapping issues. Moreover, reads originating from different isoforms or spanning complex homologous regions cannot be reliably distinguished, hindering accurate identification of novel isoforms and full-length gene fusion events [7]. In contrast to bulk short-read RNA-Seq, where paired-end reads and higher RNA input provide uniform transcript coverage and robust isoform reconstruction, single-cell protocols are impacted by sparse coverage and high dropout rates, leading to lower sensitivity and reduced recovery of transcript diversity. However, scRNA-seq studies have revealed that AS exhibits a high level of heterogeneity across cell types [8–10], and high cell-type specificity [11], particularly in cancer [12–14].

### Long-read RNA-Seq

Long-read RNA-seq (lrRNA-seq) captures full-length transcripts generating reads of up to hundreds of thousands of nucleotides in length. Consequently, lrRNA-seq has revealed many structurally diverse transcripts and novel isoforms [15]. Because long reads can span entire transcript molecules, lrRNA-seq enables the direct observation of co-occurring events within single isoforms that are often fragmented or entirely missed in short-read data. This includes the coordination between AS events and other transcript elements such as transcription start sites (TSSs) and poly(A) sites. For example, long-read studies have shown that alternative promoter usage can be tightly linked to downstream splicing patterns, with initiation at specific TSSs predisposing transcripts to exon or poly(A) site choices [16, 17].

A combination of reduced error rates, lower costs, and increased throughput has driven greater adoption of lrRNA-seq, also widely referred to as third-generation sequencing (TGS), for bulk-based and single-cell sequencing [15, 18]. Single-cell long-read RNA-seq (SCLR-seq) provides an opportunity to capture full-length isoforms at single-cell level. Unlike short-read single-cell approaches, which rely on incomplete transcript capture and isoform inference, SCLR-seq gives a comprehensive and annotation-independent view of the transcriptome, revealing the full extent of isoform diversity, AS patterns, and transcript usage in cell.

The complexity of isoform expression further fine-tunes cell identity as it contributes to the diversity of phenotypes across cells, cell types, tissues and organs among individuals [19]. With SCLR-seq, we are moving beyond differential gene expression as a source of cell diversity to differential isoform expression as differences between cell populations. These isoform level signatures allow for finer discrimination between cell subtypes, transitional states, or disease-associated phenotypes. SCLR-seq enables more detailed characterization of regulatory mechanisms such as allele-specific isoform expression, coordinated splicing, and other RNA processing events. It can also help resolve ambiguity in complex genomic loci, such as those involving bidirectional promoters or overlapping transcripts including long noncoding, which are often difficult to distinguish using short-read data.

Together, these capabilities enable SCLR-seq to provide a far more detailed and functionally relevant view of the transcriptome than previously possible. This review discusses the applications and methods of SCLR-seq with a focus on bioinformatics data analysis pipelines.

## The importance of AS in human health

Nearly all protein coding genes in the human genome undergo AS [20]. AS events facilitate the expression of transcript isoforms in a developmental, cell-specific and tissue-specific manner [21], with ~95% of multi-exon genes undergoing splicing to produce an average of 14.6 isoforms per gene [22, 23]. Different mRNA isoforms encode proteins or non-coding RNAs that may differ in their function, structure, and localization with isoforms even having opposing functions in some cases. For example, most apoptosis-related genes, such as *BCL2L1* (BCL2 Like 1) and *CASP2* (caspase-2) have multiple isoforms, some functioning to promote apoptosis while others are anti-apoptotic [24, 25], with the ratio of isoforms expressed deciding the fate of a cell. AS also plays a regulatory role in gene expression by influencing mRNA stability. It can affect the half-life of mRNA molecules by retaining or omitting certain regulatory elements, or by including a premature stop codon, causing early degradation of transcripts [26, 27]. Splicing also affects translation rates by influencing the processing and stability of mRNA, e.g. through the recruitment of exon junction complexes, which help engage translation initiation factors and enhance ribosome loading [28].

**Figure 1:**
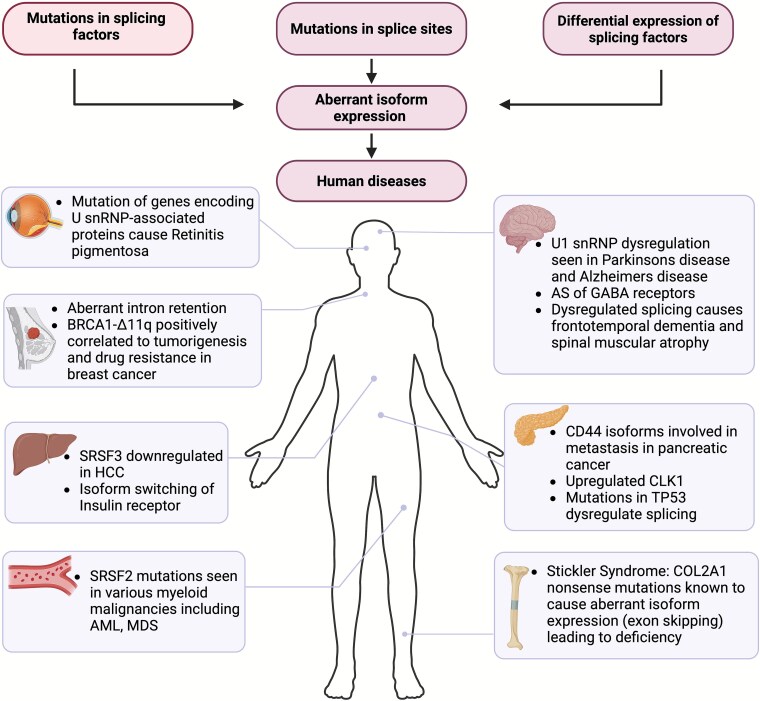
Examples of human diseases caused due to dysregulated alternative splicing**.** Created with BioRender.com.

AS is closely related to human health and often caused by genetic variants (Fig. 1). Single nucleotide variants (SNVs) such as missense mutations, stop-gain mutations, small insertions or deletions can disrupt normal splicing and cause disease [29]. These variants may interfere with splicing signals, enhancers or silencers within the pre-mRNA, leading to the production of aberrant mRNA transcripts and proteins. Such *cis*-acting variants are estimated to constitute 15%–30% of disease-causing mutations in human [30]. In contrast, SNVs that effect *trans*-regulatory elements can induce widespread splicing changes by altering splicing of many target genes; however, they are rare compared to variants in *cis* elements. Deep intronic variants can create cryptic splice sites leading to abnormal transcript isoforms [31]. Additionally, structural variants like translocations, inversions, copy number variants, and gene fusions can alter splicing by rearranging the order of exons and introns, introducing cryptic splice sites, deleting exons, and disrupting splice regulatory mechanisms [29].

Variants in consensus splice site (GU at 5′ end of the intron and AG at its 3′ end) as well as in *cis-* or *trans-* acting elements can cause or contribute to many human diseases and are collectively referred to as spliceopathies [29, 32]. For example, germline variants in *trans*-acting elements of the spliceosome were recently found to lead to converging neurodevelopmental phenotypes of developmental delay, intellectual disability, and autism [33]. Other diseases associated with aberrant splicing include bipolar disorder, schizophrenia [34], cancer [35–37], amyotrophic lateral sclerosis [38], diabetes [39], and myotonic dystrophy [40]. Splicing variants can also impact translation rates by altering binding sites for cap-binding proteins, poly(A)-binding proteins, RNA-binding proteins, or sequences upstream of the initiator codon. For example, a splicing-associated A-to-G substitution at the +1 position converted an upstream adenine-uracil-guanine (AUG) codon to a guanine-uracil-guanine (GUG) in the upstream open reading frame (uORF) of *EPHB1*, which reduced uORF translation and increased translation at the main ORF [41, 42].

Aberhart isoform expression can cause development-related pathologies such as Stickler syndrome [43], accelerate aging [44], impair functioning of the heart and brain [45]. It is also linked to various cancers and their hallmarks, including uncontrolled proliferation, metastasis, immune invasion, and oncological transformation [35, 46, 47]. For example, splice variants in CD44, a transmembrane glycoprotein expressed on the surface of many cells, are known to be involved in tumor metastasis in pancreatic cancer and breast cancer [48]. Missense mutations in *TP53* (tumor suppressor 53) have been associated with the dysregulation of AS in pancreatic cancer [49]. It has also been noted that the expression of factors involved in pre-mRNA splicing is most altered in cancer [50]. By example, SRSF3 is a splicing factor (SF) responsible for AS of target genes involved in glucose and lipid metabolism, in addition to roles in cytoskeleton organization and cellular growth. It is downregulated in hepatocellular carcinoma (HCC), and its knockdown in mouse hepatocytes promotes the development of metastatic HCC [51]. SRSF2 is an RBP that plays important roles in splicing of mRNA precursors. *SRSF2* mutations are the most common splicing machinery mutations identified in various myeloid malignancies like acute myeloid leukaemia, myelodysplastic syndrome, chronic myelomonocytic leukaemia, and myeloproliferative neoplasms [52].

### Other sources of isoform diversity

In eukaryotic cells, isoform diversity is substantially expanded by context-dependent alternative RNA processing events. These include the differential use of TSSs, splice sites, and polyadenylation sites [53]. Among these, alternative polyadenylation plays a critical role in shaping transcript isoforms by altering 3′ untranslated region length or generating distinct protein-coding variants thereby influencing mRNA stability, localization, and translational efficiency. More than half of human genes are estimated to undergo alternative 3′-end processing [54].

In addition to canonical processing, transcriptomic complexity is further enhanced by posttranscriptional RNA modifications. The most prevalent of these is N6-methyladenosine (m6A), the methylation of adenosine residues. m6A modifications impact multiple aspects of RNA metabolism including splicing, nuclear export, stability, and translation thereby adding an additional regulatory layer that contributes to isoform and functional diversity [55].

Further complexity arises from RNA editing, particularly adenosine-to-inosine (A-to-I) editing mediated by Adenosine Deaminase Acting on RNA (ADAR) enzymes, which can recode transcripts, alter splice sites, or modulate RNA structure [56]. Transcriptional readthrough and the formation of chimeric transcripts often spanning adjacent genes also expand the isoform landscape, especially in certain disease contexts, such as cancer [57]. Complex gene architectures including overlapping or antisense transcripts contribute to transcriptomic ambiguity that is difficult to resolve with short-read methods but can be clarified using long-read sequencing [58, 59].

## TGS platforms for SCLR-seq

Pacific Biosciences (PacBio) and Oxford Nanopore Technologies (ONT) are the two prominent platforms for TGS that are increasingly adopted in SCLR-seq experiments. PacBio sequencing provides high accuracy, but its substantial setup and sequencing costs restrict its use. In contrast, ONT sequencers are affordable, provide high throughput, but come with a higher sequencing error rates impacting base call accuracy [60].

PacBio’s single-molecule real-time (SMRT) sequencers (Sequel, Sequel II, Sequel IIe, Revio, and Vega) detect nucleotides as they are incorporated in real-time by monitoring fluorescence pulses emitted as DNA polymerase synthesizes a complementary strand within nanophotonic zero-mode waveguides [61]. Sequel II can produce up to 24 Gb of data per day, whereas the Revio system can produce up to 360 Gb data per run [62]. Both systems support circular consensus sequencing, in which a single DNA molecule is repeatedly sequenced in a circularized format to generate a highly accurate consensus read. PacBio claims that using this method leads to read accuracies of 99.9% and 99.95% in their Sequel II and Revio sequencers, respectively. However, the Sequel II and Sequel IIe systems are no longer available for sale.

When using 8M SMRT Cells (containing 8 million zero-mode waveguides), up to 2 million HiFi reads can be obtained. The MAS-Iso-Seq approach developed by Al’Khafaji *et al*. reaches nearly 40 million HiFi reads per run on the Sequel IIe sequencer [63]. PacBio adopted MAS-Iso-Seq in their Kinnex library preparation kits, which produce sequencing ready SMRTbell libraries for use on either Revio or Vega systems, which are their latest models in PacBio’s long-read sequencer fleet. Revio can generate up to 80–100 million reads per run and has 20× the compute power of the Sequel IIe system, whereas Vega is a benchtop sequencer that can generate 50–60 million reads per run.

ONT’s sequencing device range includes the portable MinION, and the more stationary GridION, and PromethION, which can be used with one or more different types of flow cells including MinION (2048 pores), Flongle (512 pores), and PromethION (10 240 pores). A single PromethION flow cell can generate up to 250 million reads theoretically, making it a powerful tool for high-throughput applications [64]. However, such yields are not typical in practice due to factors such as library quality, sample complexity, and flow cell performance. Nanopore sequencing used to encounter high error rates; however, with recent advancements in flow cell chemistry and improvements in basecalling algorithms, ONT reported accuracies of up to 99% [65].

### SCLR-seq library preparation methods

Like short-read scRNA-seq, libraries for SCLR-seq can be prepared using droplet based, plate based, or microwell-based methods. Multiple 10× Genomics kits have demonstrated compatibility with both PacBio [66, 67] and ONT [68, 69] protocols (Table 1). The 10× Genomics Chromium platform for single-cell analysis can facilitate capture of 10 000 cells per library and enable rare cell type discovery; however, it suffers from a high dropout rate, especially for genes with low expression [70]. Two recently introduced protocols for SCLR-seq, ScISOr-Seq [67] and ScNaUmi-seq [69], adopt 10× Genomics’ Chromium kits to barcode cells and to split them for complementary long- and short-read sequencing. Both protocols use the short reads for error correction and guiding barcode assignment of the long reads. Similarly, the FLT-Seq library preparation method uses a modified Chromium 10× droplet-based workflow in which a subset of gel beads-in-emulsions (GEMs) (e.g. 10%–20%) is diverted for long-read sequencing, while the remainder proceeds with standard short-read sequencing. This “hybrid subsampling” strategy allows leveraging the high throughput and low cost of short-read sequencing for cell clustering and barcode assignment, while still gaining full-length isoform data for a subset of cells [71]. The RAGE-seq [72] protocol, introduced by Singh *et al*. in 2019, in addition to sequencing both short and long reads, performs a targeted capture prior to sequencing to improve depth of coverage. However, a distorted distribution of CBs and UMIs was observed due to polymerase chain reaction (PCR) cycles and error rates of ONT. Rapid Capture Hybridization Sequencing is a targeted capture hybridization method applied to (single-cell) full-length cDNA, where probes are used to enrich transcripts of interest, which are then sequenced with long reads [73]. Long-read targeted scRNA-seq is a more recent technique to combine targeted long-read sequencing of selected transcripts with the retention of single-CBs/UMIs, using a custom probe panel, thereby focusing sequencing resources on molecules of interest (e.g. low-abundance or functionally important isoforms) [74]. Single cells isolated by Drop-seq are sequenced on an ONT platform in a protocol termed scCOLOR-seq [64], which aims at reducing errors in barcodes. This protocol incorporates bi-nucleotide repeats in barcode sequences which enables detection and correction of sequencing errors in barcodes. It demonstrated significant improvement in barcode accuracy across different cell types, yielding reliable isoform detection, proving to be a robust method to perform quantitative long-read transcript sequencing on large numbers of cells. Nonetheless, it requires customized synthesis of gel-beads and prior knowledge of true barcodes [64].

**Table 1:** Available single-cell kits and their compatibility with ONT and PacBio TGS platforms

**Available kits**	**Company**	**Compatibility with ONT**	**Compatibility with PacBio**
Chromium Next GEM Single Cell 3′ Kit v3.1	10× Genomics	Validated and supported	Validated and supported
Chromium Next GEM Single Cell 5′ Kit v2	10× Genomics	Validated and supported	Validated and supported
Chromium Next GEM Single Cell 5′ Kit v2—V(D)J library	10× Genomics	Currently being tested	Incompatible
Chromium GEM-X Single Cell 5′ Kit v3	10× Genomics	Not tested	Compatible with Kinnex kit, not tested for MAS-Seq kit
Chromium GEM-X Single Cell 3′ Kit v4	10× Genomics	Not tested	Not tested
Chromium Next GEM Single Cell Multiome ATAC + Gene Expression	10× Genomics	Currently being tested	Compatible with both kits
Chromium Next GEM Single Cell ATAC Kit v2	10× Genomics	Not tested	Incompatible
Chromium Next GEM Single Cell Fixed RNA Sample Preparation Kit	10× Genomics	Incompatible	Incompatible
Evercode Whole Transcriptome v3	Parse Biosciences	Currently being tested	Compatible, tested in-house
ScaleBio Single Cell RNA Sequencing Kit v1.1	Scale Biosciences	Not tested	Not tested/not compatible (not specified)
ScaleBio™ Single Cell Methylation Kit	Scale Biosciences	Not tested	Not tested/not compatible (not specified)
Illumina Single Cell 3′ RNA Prep	Fluent BioSciences (Illumina)	Not tested	Tested with MAS-seq

Smart-seq2 [75] is a plate-based method that was initially developed for full-transcript coverage on Illumina next-generation sequencing platforms. Different research groups have built on the Smart-seq2 protocol to enable efficient sequencing on TGS platforms. The R2C2 (Rolling Circle to Concatemeric Consensus) protocol uses the Tn5Prime method to prepare libraries, which is a modification of the smart-seq2 method that uses a distinct template switch oligo (TSO) containing 7-nt sample indexes during reverse transcription [76]. By using the circular consensus principle, the protocol achieves ~96% sequencing accuracy with ONT-based long-read sequencing. Plate-based methods limit the number of cells that can be sequenced: R2C2 sequenced 1–96 cells, while SCAN-seq2 [77], another plate-based method compatible with ONT sequenced 96–960 cells. However, it is possible to capture more cells by processing more plates in parallel, although this increases cost and handling complexity.

Microwell-based methods such as Parse Biosciences’ Evercode™ have also been used with PacBio [78] and ONT [79] (Table 1). Microwell-based methods use combinatorial indexing and alleviate the use of microfluidic devices. They have higher throughput, but lengthy hands-on time, which could lead to inconsistencies stemming from human error [80].

## Bioinformatics analysis of SCLR-seq data

**Figure 2:**
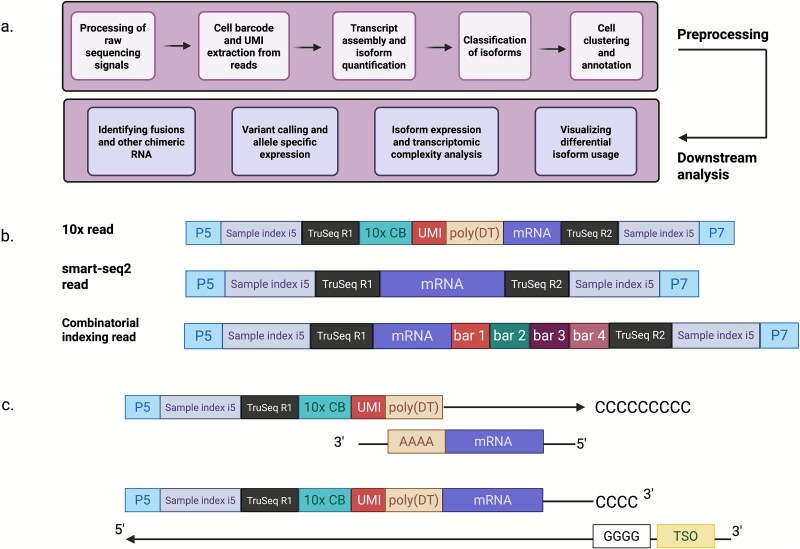
SCLR-seq data handling. (a) Basic steps involved in data analysis of SCLR-seq data. For a more exhaustive list see Table 2 and long-read-tools.org [82]. (b) Sequencing read structures of different single-cell library preparation methods. Most droplet-based methods such as 10× Genomics utilize a CB and a UMI. Methods that use combinatorial indexing such as Parse Biosciences incorporate four barcodes for a read. The combination of these barcodes helps differentiate between cells and genes. (c) The architecture of single-cell long-reads. In the 3′ kit of 10×, cDNA is sequenced using the poly-A tail; thus, it is sequenced from the 3′ to 5′ direction. The parent strand is generated by using a template switch oligo (TSO). The TSO causes the reverse transcriptase enzyme to switch from transcribing the RNA template sequence to transcribing the TSO sequence, generating the complementary cDNA strand. Created with BioRender.com.

Analysis of SCLR-seq data involves a series of computational steps, each designed to interpret the transcriptome at single-cell resolution using full-length reads. These typically include: (i) extraction of CBs and UMIs, (ii) read alignment and transcriptome assembly, (iii) quantification of genes and isoforms, (iv) cell-type identification and clustering, and (v) downstream analyses such as isoform classification, differential isoform usage, and differential splicing (Fig. 2a). Downstream analyses like AS analysis and isoform classification can be performed using existing tools for single-cell [81] or long-read data [82]; however, functionality might be limited when applied to SCLR-seq data. While many of these steps are well-established for bulk or short-read scRNA-seq, SCLR-seq introduces unique challenges. These include higher per-read error rates, barcode or UMI loss, lack of a defined read structure (e.g. 3′ bias), and difficulties in assigning transcript boundaries due to read truncation or chimera formation. As a result, conventional single-cell or long-read tools often require adaptation or entirely new methods for accurate SCLR-seq analysis.

A growing number of tools and pipelines have been developed to support SCLR-seq analysis. These range from end-to-end workflows, which automate most of the pipeline using tools focused on individual tasks such as demultiplexing, alignment, or isoform quantification. The sections below follow the typical analysis workflow and, for each step, outline the key conceptual goals, challenges unique to SCLR-seq, and tools currently available. Both PacBio and ONT also provide their own workflows to perform end-to-end analysis. This section will discuss current trends in methods used to interpret SCLR-seq data.

### Processing of raw sequencing signals

Raw sequencing data generated from ONT platforms are stored in POD5, FAST5, or SLOW5 [83] format, which contains the squiggle signal produced via disruption in ionic current flow as cDNA fragments traverse nanopores embedded in a membrane. The raw signal is then analyzed by a basecaller software that uses deep learning models to determine the nucleotide sequence encoded in each read. ONT, developed its own high-accuracy basecaller *dorado* (github.com/nanoporetech/dorado), which stores sequencing reads in FASTQ files and has built-in functions for demultiplexing, as well as adapter- and poly-A trimming; however, it cannot be used to extract CBs and UMIs.

PacBio HiFi reads, which include fluorescent pulses, need to be divided into separate segmented reads (S-reads) that correspond to the original cDNA sequences. This can be done on PacBio’s analysis software *SMRT Link* (www.pacb.com/smrt-link/). Read segmentation includes converting fluorescent pulses into continuous long reads, then into subreads which are stored as unaligned BAM files. This is finally converted to a continuous circular read, which is compatible for use in PacBio’s downstream *Iso-Seq* workflow.

### CB and UMI extraction from reads

Accurate identification of reads in scRNA-seq data relies on accurate recovery of CBs and UMIs, which are incorporated during library preparation. In droplet-based methods like 10× Genomics, each cDNA molecule is tagged with a CB to indicate its cell of origin and a UMI to uniquely label that original molecule and help identify and collapse PCR duplicates (Fig. 2b and c).

In short-read platforms, CB and UMI sequences are predefined, and a whitelist of known barcode sequences is provided, enabling straightforward extraction and error correction. Barcode recovery becomes challenging when sequenced in long-read platforms with several complications: (i) high error rates in long-read platforms complicate sequence matching and increase false positives. (ii) Barcodes may be partially or incorrectly sequenced, leading to loss of whitelist matches. (iii) Chimeric reads. Chimeric cDNA reads can be identified if they contain: (a) two adjacent internal poly-A signals with more than 200 nucleotides between them and are flanked by an adapter, (b) one internal adapter with an adjacent template switch oligo (TSO), or (c) two adjacent TSOs. TSOs are adapters used to generate the template strand from the cDNA (Fig. 2b).

Inaccurate detection of barcodes and UMIs has a direct impact on isoform discovery and quantification. It can cause false detection of spurious new isoforms and inaccurate quantification. To prevent these errors, most studies sequence the same cDNA library with both short-read and long-read methods. Short reads are used to construct reference barcodes and aid in barcode recovery from long reads. Tools like *SiCeLoRe* [69] and *scTagger* [84] allow demultiplexing of single-cell long reads from accompanying short reads. However, SCLR-seq-specific tools now facilitate barcode detection without the need for accompanying short reads.

Several tools have been developed for CB and UMI detection. ONT’s *epi2me-labs/wf-single-cell* pipeline (github.com/epi2me-labs/wf-single-cell) supports barcode extraction, UMI parsing, and deduplication, using expected sequence motifs like poly-A tails and 10× adapters to orient reads. Similarly, *BLAZE* [85] identifies the poly-T signal and 10× adapter to extract CB and UMI tags directly from raw reads, saving them in a format compatible with downstream tools like *FLAMES* [71]. *BLAZE* does not require a known whitelist and can tolerate sequencing noise by applying a configurable edit distance during correction. *SiCeLoRe* further addresses the issue of chimeras by scanning for repeated adapter or poly-A motifs within long reads and splitting them into putative single-transcript molecules. This is particularly important in full-length sequencing protocols that may capture multiple cDNAs within a single read. *Flexiplex* [86], like *BLAZE*, works without known barcodes and also splits chimeric reads like *SiCeLoRe*. All these tools work with UMI-based single-cell protocols. PacBio’s *Iso-Seq* workflow [87] uses primers and the poly-A tail to orient the read into a 5′–3′ direction, prior to removing them using *lima*. CBs and UMIs are then extracted and corrected using a reference list, followed by read deduplication based on corrected values. Microwell-based protocols like Parse Biosciences [88] that use combinatorial indexing have their own custom pipeline for barcode handling. No independent tool is available yet to facilitate this. A comparison of tools for barcode and UMI extraction, correction, and chimera handling in SCLR-seq is shown in Table 3.

UMI error correction strategies include clustering UMIs within a fixed edit distance. Edit distance is defined as the number of nucleotide changes (insertions, deletions, or substitutions) required to match two sequences. An appropriate threshold depending on single-cell protocol/sequencing platform is used to offer a good balance between efficiency and specificity to allow minor corrections accounting for sequencing errors. Read inflation can also occur when the number of UMIs is overestimated in cases of high gene expression and high PCR amplification. *Longcell* [89] utilizes PCR duplicates to correct truncation and mapping errors as a UMI recovery method and applies an iterative clustering procedure to cluster putative UMI sequences. A recent study used clustering-based methods (e.g. *starcode*) to group similar barcodes and correct them to consensus sequences, while UMIs were corrected using graph-based models that incorporated cDNA length and sequence similarity to collapse amplification artifacts [90].

### Transcript assembly and isoform quantification

Transcript assembly and isoform quantification are central to understanding transcriptomic diversity at single-cell resolution. The aim of this step is to reconstruct full-length transcripts or isoforms from mapped reads and assign expression levels to each isoform in individual cells. While known challenges associated with both single-cell and long-read sequencing persist, including data sparsity, high dropout rates, sequencing errors and read length bias, SCLR-seq introduces additional complexities: (i) sparse coverage due to insufficient reads per cell makes transcript reconstruction more uncertain. (ii) The accurate retention and handling of CBs and UMIs throughout the analysis are critical, as any loss or misassignment can lead to incorrect cell and transcript quantification. (iii) Isoform ambiguity arising from clustering approaches used or from sequence features, such as homology, complicates accurate isoform quantification.

For alignment, the long-read mapper *Minimap2* is widely used [91]. It supports spliced alignment and retains CB and UMI (UB) tags in the BAM output files, making them compatible with downstream isoform assembly tools. While tools such as *Bambu*, *StringTie2*, *TALON*, *FLAIR*, and *IsoQuant* have been extensively benchmarked for isoform discovery and quantification in bulk long-read datasets, they are not inherently designed for SCLR-seq, as they cannot track reads back to individual cells without additional preprocessing [92–95]. Even with barcode-aware preprocessing steps, these tools remain UMI-unaware, meaning they cannot perform accurate molecular deduplication at the isoform level. scRNA-seq tools that are UMI-aware such as *UMI-tools* or *Alevin* are built for short-read data and do not support full-length isoform reconstruction [96, 97]. The need for tools and pipelines that simultaneously support long-read isoform discovery and UMI-aware quantification highlights a major computational challenge in SCLR-seq analysis.

There is a growing need for isoform-aware UMI deduplication methods as well. Isoform discovery algorithms usually cluster similar isoforms into groups and collapse each cluster into a single representative isoform. While effective for reducing noise, this approach risks collapsing distinct isoforms into a single dominant isoform, leading to inaccurate isoform quantification. Some methods also include an Expectation–Maximization algorithm to estimate transcript expression levels when reads map to multiple isoforms or use splice graph reconstruction to infer isoform models based on exon connectivity. Tools that consider isoform-level structure during deduplication are essential to preserve transcriptomic resolution in SCLR-seq data (Table 4).

Several standalone tools and pipelines have been developed specifically for SCLR-seq analysis workflows. Each tool varies in its compatibility with platforms, barcode requirements, and transcript quantification accuracy. While most require a BAM file already tagged with barcodes and UMIs, a few pipelines offer fully integrated workflows from raw FASTQ to expression matrix (Table 2). Some popular pipelines include *FLAMES*, *SiCeLoRe*, *bambu-clump* [98], *nanoseq* [99], and *scNanoGps* [100] (Table 2). *FLAMES* is an end-to-end analysis pipeline for ONT-based SCLR-seq data. It integrates *BLAZE* or *flexiplex* for barcode extraction and uses *bambu* for transcript assembly and *oarfish* [101] for transcript quantification in cells. It generates cell-by-isoform expression matrices and supports multiple UMI-based single-cell protocols. *SiCeLoRe* processes ONT reads by orienting them based on adapter sequences and detecting chimeric cDNAs, which are split and assigned to transcripts. It reconstructs isoforms per cell and produces expression matrices but can be sensitive to read sparsity. *Bambu-clump*, included in the *bambu* package, enables transcript discovery at the bulk and pseudo-bulk level; it can be used for quantification of fusion transcripts, and it returns full-length read support for all transcripts to support cell-marker validation [98]. Data sequenced using Parse Biosciences’ single-cell methods can currently only be preprocessed using Parse’s own custom pipeline. For PacBio Iso-Seq data, the pipeline typically involves *Cupcake* to collapse full-length reads and *IsoQuant* to quantify isoform expression. With appropriate barcode tagging, this approach can also be adapted to single-cell analyses. 

**Table 2:** Bioinformatics tools specific for single-cell long-read sequencing

**Tool**	**Application**	**Technology**	**TGS platform**	**Key features**	**References**
**End-to-end pipelines**					
FLAMES	Transcript quantification	10× /UMI based	Predominantly for ONT	Isoform discovery, splicing analysis, and mutation detection	[71]
SiCeLoRe	Transcript, gene, and junction quantification	10× /UMI based	Predominantly for ONT	Barcode and UMI identification, isoform quantification, mutation detection	[69]
nanoseq	End to end for SCLR-seq	10×/UMI based	ONT	Quality control, transcript discovery and quantification, differential expression analysis RNA fusion, and RNA modification detection	[99]
EPI2ME Labs / wf-single-cell	ONT’s pipeline for SCLR-seq	10×	ONT	Fused read splitting, CB and UMI extraction and correction	github.com/epi2me-labs/wf-single-cell
scywalker	End to end	10×/UMI based	ONT	Short-read data are optional, pseudo-bulk isoform counts	[102]
Bambu-clump	Transcript discovery	10×/UMI based	ONT	Splice junction-based transcript discovery	[98]
Parse custom pipeline	Demultiplex and preprocessing	Parse single cell	ONT	Deals with multiple barcodes generated due to combinatorial indexing	[88]
Isoseq3	End-to-end pipeline by PacBio	PacBio single cell	PacBio		[87]
scNanoGPS	Preprocessing quantification and variant calling	10×	ONT	Tested for single-cell and single-nucleus	[100]
					
**Identification of cell barcodes and UMI**					
scTagger	Match cellular barcodes from long-reads and short-reads	10×	ONT (needs short reads)	Nonzero edit distance matching	[84]
BLAZE	Linux, python	10× /UMI based	Predominantly for ONT	Fast and easy demultiplexing of large-scale SCLR-seq	[85]
Flexiplex	Demultiplex and split chimera	10×/UMI based	Predominantly for ONT	A stand-alone tool that identifies cell barcodes and UMIs	[86]
Longcell	UMI deduplication, isoform quantification, and differential splicing analysis	10×/UMI based	Predominantly for ONT	UMI, read mapping correction, and truncation errors without relying on existing isoform annotations	[89]
**Standalone isoform quantification**					
Isosceles	Isoform assembly and analysis	10× /UMI based	Predominantly for ONT	Isoform detection and quantification across single-cell, pseudo-bulk, and bulk resolution	[103]
Lr-kallisto	Transcript discovery and quantification	10× and Parse	Predominantly for ONT	Pseudoalignment for long-reads	[104]
SCOTCH	Isoform assembly and analysis	10× /UMI based and Parse	Predominantly for ONT	Isoform detection and quantification	[105]
Oarfish	Isoform quantification	UMI based	ONT	Isoform quantification	[101]
**Other**					
LongSom	Somatic variant calling	10×	Only tested for PacBio	Distinguishes somatic SNVs from noise and germline polymorphisms	[106]
SlSim	Simulate single-cell long-read FASTQ files	10×	ONT	Generates perfect and erroneous reads	github.com/youyupei/SLSim
AsaruSim	Simulate SCLR-seq data	10×	ONT	Workflow to simulate count matrix and sequences	[107]
CTAT-LR-Fusion	Fusion caller for SCLR-SEQ data	10×	PacBio (tested on simulated ONT)	Detect fusion transcripts	[108]
ScisorWiz	Visualizing differential isoform expression	any	both	Differential isoform expression through various clustering methods	[109]
ScIsoX	Transcriptomic complexity analysis	Any	Both	Metrics that capture transcriptomic complexity across biological scales	[110]
scisorseqr	Differential splicing analysis	10×/UMI based	ONT and PacBio	Barcode deconvolution, mapping, and filtering of high confidence, full-length spliced reads.	[111]
Isopod	DTU analysis	10×/UMI based	ONT and PacBio	Differential transcript usage analysis in R	https://github.com/michael-nakai/isopod

**Table 3:** Tools dealing with barcodes

**Tool**	**Application**	**Standalone tool**	**Isoform clustering**	**Correct cell barcodes**	**Correct UMIs**	**Split chimeras**	**Key feature**	**References**
BLAZE	Barcode extraction and demultiplexing	Yes	No	Yes	Yes	No	Fast and easy demultiplexing of large scale SCLR-seq	[85]
Flexiplex	Demultiplexing with error-tolerant barcode/UMI search	Yes	No	Yes	Yes	Yes	Handles split chimeras, error-tolerant demultiplexing	[86]
SiCeLoRe	Barcode/UMI assignment	Part of pipeline		Yes	Yes	Yes	Handles chimeras, accurate assignment of cell barcodes and UMIs	[60]
Longcell	Accurate barcode recovery, iterative clustering of UMIs	Part of pipeline		Yes	Yes	No	Iterative clustering of UMIs, accurate barcode recovery	[89]
scTagger	Match cellular barcodes from long-reads and short-reads	Yes	No	Yes	Yes	No	Nonzero edit distance matching	[84]

All tools are for UMI-based single-cell technologies such as 10× and sequenced on nanopore sequencers.

**Table 4:** Comparison of tools for isoform discovery and quantification

**Tool**	**Platform**	**Transcript assembly method**	**Strengths**	**Limitations**	**References**
FLAMES	ONT	Semi-supervised isoform discovery and quantification	End-to-end, option to use other tools such as bambu and oarfish	Biased novel isoform, high reads discarded	[71]
SiCeLoRe	ONT	UMI-guided error-correction by consensus generation	Handles chimeras well, end-to-end	High reads discarded, underperforms in highly sparse datasets	[69]
wf-single-cell	ONT	Directed graph algorithm	UMI guided isoform discovery, GUI available for ease of use	Limited benchmarking, memory intensive	github.com/epi2me-labs/wf-single-cell
SCOTCH	ONT/PacBio	Sub-exon identification with dynamic thresholding	Platform-agnostic; no short-read needed	Requires high-quality BAM with tags, newer tool with less validation	[105]
Isosceles	ONT	Splice graph-based isoform reconstruction	High fidelity isoform resolution; DTU support	Specific to ONT; newer tool with less validation, non standard naming of tags	[103]
Lr-kallisto	Predominantly ONT	Pseudo-alignment for long-reads	Not memory intensive, tested for single-nuclei too	Accuracy goes down with high sequencing error rate	[104]
Bambu-clump	ONT	Built on bambu’s splice junction matching algorithm	Preprocessing pipeline, internal demultiplexing	Newer tool with less validation	[98]
Cupcake + IsoQuant	PacBio	Collapsing and filtering	Mature workflow for Iso-Seq data	Not single-cell-specific without CB tagging	[87]
LongPreCell (preprocessing to Longcell)	ONT	UMI-based correction of reads by taking consensus	When coupled with IsoQuant gave better outcome in independent study	Newer tool with less validation	[89]
nanoseq	ONT	Integrates previous tools such as bambu, stringtie2	Preprocessing and downstream analysis	Newer tool with less validation as whole pipeline	[99]
oarfish	ONT	Probablistic method, not UMI aware	Quick, not demanding computationally	Requires preprocessing, no UMI correction	[101]
scNanoGPS	ONT	Deconvolution of error-prone long-reads	Mainly for downstream analysis such as genotype-phenotype correlation	ONT specific, limited benchmark	[100]
bambu/ StringTie2/TALON/FLAIR	ONT/PacBio	Various (annotation-guided, graph-based)	Well-benchmarked for bulk	Cannot natively assign to cells	[92, 93]

For more details authors suggest going through external benchmark papers such as Hamraoui et al. [112].

Standalone tools for isoform discovery and quantification have the advantage of flexibility allowing user customization. *SCOTCH* is a modular toolkit compatible with ONT, PacBio, and a variety of single-cell platforms (e.g. 10×, Parse Biosciences). It reconstructs isoforms directly from long reads and addresses ambiguous mapping challenges through sub-exon identification with dynamic thresholding and read mapping scores [105]. A pseudoalignment for long reads is used *in lr-kallisto*, which is robust to error rates of sequencing platforms [104]. *Isosceles* is an R-based package for ONT data that uses a splice graph–based approach to infer isoform structure, offering high fidelity in transcript resolution [103]. It supports downstream analyses like differential transcript usage (DTU) and clustering at the single-cell, pseudo-bulk, and bulk levels.

Following isoform assembly and quantification, the next step involves classification and quality control of isoforms to remove artifacts and categorize transcript novelty. This is essential to ensure accurate downstream analyses.

### Classification of isoforms

Once isoforms have been assembled and quantified, tools such as *SQANTI3* perform detailed classification and filtering. This step uses transcript structural features and external annotations to distinguish high-confidence isoforms from technical artifacts. These artifacts include transcripts with low coverage, RT-switching (reverse transcriptase template switching), and intra-priming (with adenine stretches downstream of the 3′ end). *SQANTI3* [113] is currently one of the most comprehensive and widely adopted methods for classifying and filtering isoforms from long-read sequencing data.

Isoform splice junctions are compared with known splice junctions to classify isoforms into the following categories: full splice match, incomplete splice match, novel in catalog (NIC), and novel not in catalog (NNIC). The ordered list of all introns in a read, also called an intron chain, is matched to an annotated transcript. If a read has a novel intron chain but uses known splice sites, it is classified as NIC. If a single splice site on the read differs from known splice sites, it is classified as NNIC [113]. Additionally, if an isoform maps to two different genes, it is classified as a fusion. Depending on which region of the genome a novel transcript maps to, it can be classified as intergenic, genic intron (if located within the boundaries of an annotated intron,) or genic genomic (partial overlap with known gene). A read can also map to the antisense strand of a transcript and is classified as “antisense.” As an antisense classified transcript also contains a poly-A tail, it is unlikely that it is a reverse complement. *SQANTI3* can also be used to assess the reliability of isoforms by annotating with publicly available Cap Analysis of Gene Expression peaks and poly(A) sites to filter out PCR artifacts. *SQANTI3* generates an annotated isoform file in gff3 format which is compatible with downstream splicing analysis tools such as *IsoAnnotLite* and *tappAS* [114]. The *IsoAnnot* pipeline integrates coordinate-defined functional annotations at the RNA and protein levels, derived from public databases and sequence-based prediction tools. *tappAS* uses isoform-resolved annotation of coding and non-coding functional domains, motifs, and sites, in combination with novel analysis methods to interrogate different aspects of the functional readout of transcript variants and isoform regulation.

Following isoform classification and filtering, the resulting isoform expression matrices enable many downstream analyses. An immediate next step is usually cell clustering and annotation. Accurate clustering is essential to reveal cellular heterogeneity and assign biological identities to individual cells or cell populations.

### Cell clustering and annotation

Read counts must be normalized to be comparable, scaled, and filtered prior to cell clustering. Similar cells are clustered together based on their gene expression profiles or other molecular signatures. Cell clusters are visualized in a 2D map using dimensionality reduction techniques like t-distributed stochastic neighbor embedding [115] or uniform manifold approximation and projection [116]. Cell types are identified by mapping markers expressed in a cell to an annotated cell atlas or a reference set. Assigning biological identity to an individual cell or a distinct cluster can be done manually using domain knowledge or computationally using machine learning models. For example, *Seurat’s* FindMarkers function uses differential expression analysis to identify biomarkers that are defining clusters [117]. It heavily relies on existing biological knowledge of known marker genes and involves subjective decision-making, such as choosing the number of clusters (resolution) [118]. Some examples of automated cell annotation tools include *SingleR* [119] and scPred [120].

While tools such as *Seurat, Scanpy, SingleR*, and *scPred* have been widely adopted for cell clustering and annotation in short-read scRNA-seq datasets, they are not inherently designed to handle isoform-resolved data or take advantage of the full-length transcript information that SCLR-seq provides. These methods typically rely on gene-level expression matrices, which may mask critical isoform-level regulatory differences, particularly in systems where AS plays a major role. When applied to SCLR-seq data, this can lead to suboptimal clustering, as transcripts with distinct functions may be collapsed into a single gene-level expression signal. As such, there is a clear need for new tools or adaptations of existing methods that can fully exploit the isoform resolution offered by SCLR-seq for high-resolution cell-type classification, trajectory inference, and cell-state modeling.

Also, there is currently a lack of standardized reference databases for isoform-level cell-type markers, hindering automated annotation using full-length isoforms. To fully harness the potential of SCLR-seq, there is a need for new computational tools and adaptations of existing ones that operate directly on isoform-resolved expression matrices. Such methods would enable more accurate modeling of cellular heterogeneity, especially in contexts where AS plays a key regulatory role.

### Isoform expression and transcriptomic complexity analysis

Once cells have been clustered and annotated, the focus shifts to understanding the transcriptomic diversity that underlies distinct cell types or states. This involves quantifying isoform-level expression and examining patterns of AS, DTU, and other transcriptomic complexities across individual cells. SCLR-seq allows the identification of differential and cell-type-specific splicing events and the quantification of relative isoform abundance. The percent spliced-in (PSI) metric refers to the ratio between reads reflecting the inclusion of a specific sequence element (exon, intron, alternative splice site) and the sum of reads reflecting the inclusion and exclusion of these elements: Ψ = inclusion reads/(inclusion reads + exclusion reads). PSI values can elucidate all AS event types, including exon skipping, mutually exclusive exons, alternative 3′/5′ splicing, and intron retention. For example, a PSI of 1 indicates constitutive exons that are included in all transcripts and never removed. PSI values below 1 imply reduced inclusion of alternative exons and denote the percentage of transcripts that contain the exon compared to the total transcript population.

Tools to assess differential isoform expression and AS in bulk RNA-seq data such as *SpliceWiz* [120], *rMATS* [121], and *SUPPA2* [122] are well known. Tools specific to assessing differential splicing at the single-cell level include *MARVEL* [123], *BRIE2* [124], *Expedition* [125], and *VALARIE* [126]*.* Differential isoform expression analysis of SCLR-seq data can be performed with the R-package *scisorseqr* [111], which supports sequencing reads from both Nanopore and PacBio platforms and integrates tools for barcode deconvolution and alignment. However, it is not a preprocessing pipeline and requires that CBs be annotated to cell types before use. There are currently no gold standard methods available for differential splicing and isoform expression analysis specifically for SCLR-seq data.

Differential isoform expression, also referred to as DTU, can reveal cues into development and disease. For instance, Leung *et al*. identified a switch of dominant isoform expression of the *RTN4* gene between adult and fetal brain cortex in humans [127]. *RTN4* encodes a neurite outgrowth inhibitor specific to the central nervous system. DTU analysis reveals the relative abundance of different isoforms of a gene, whereas differential transcript expression (DTE) looks at differences in the combined expression of all isoforms of a gene. DTU informs the proportion of the isoform change between two conditions, whereas DTE informs how much the total expression of the gene has changed between the two conditions. *Sierra* [128] and *DTUrtle* [129] are tools for DTU analysis from scRNA-seq data, while *Isosceles, Isopod (*https://github.com/michael-nakai/isopod*)*, and *FLAMES* can be used for DTU analysis from single-cell long reads.

SCLR-seq can thoroughly enhance AS analysis, RNA editing, and allelic-specific expression analysis at the single cell level. In addition, long reads cover information on TSS usage and the poly A site usage, patterns of which are evolutionarily conserved and sometimes perturbed in disease [130].

Lastly, transcriptomic complexity analysis describes the systematic examination of global and cell-type-specific isoform expression patterns arising from AS. *ScIsoX* is currently the only framework for multidimensional transcriptomic complexity analysis that uses novel metrics such as (i) inter- and intra-cellular isoform diversity, (ii) intra-cell-type heterogeneity, or (iii) inter-cell-type specificity to capture isoform expression patterns across biological scales. *ScIsoX* operates on isoform count matrices and provides a visualization ecosystem that supports transcriptomic complexity analysis [110].

### Identifying fusions and other chimeric RNA

Fusion transcripts arise from gene fusions but can also arise without any DNA rearrangement, e.g. when adjacent genes are transcribed together (transcriptional readthrough) or unconventional splicing events fuse exons from two different transcripts (*trans*-splicing) [131]. Fusion transcripts can also have clinical implications such as the *BCR/ABL1* fusion, a known disease driver in chronic myelogenous leukemia, which is used for diagnosis and is targeted with tyrosine kinase inhibitors [117]. Tools for either scRNA-seq or lrRNA-seq data such as *JAFFAL* [132] and *LongShot* [133] can be used to identify fusions and other chimeras. *SQANTI3* [113] also annotates fusion isoforms. *CTAT-LR-fusion* is another tool that detects fusion transcripts from lrRNA-seq data at both bulk and single-cell levels, with the option of including accompanying short reads in the analysis [108].

Fusions and alternatively spliced transcripts can generate neoepitopes, and their analysis can enhance our understanding of tumor immunogenicity and inform the design of personalized immunotherapies [7, 134, 135]. Kahles *et al*. identified major histocompatibility complex I (MHC I) epitopes derived from AS events in breast and ovarian cancers using the The Cancer Genome Atlas (TCGA) immunopeptidomics database, finding them to be more abundant than epitopes derived from single-nucleotide variants [136].

### Variant calling and allele-specific expression

SCLR-seq provides a unique opportunity to perform somatic variant calling in cells using long reads. Long reads enable the direct observation of multiple variants on the same transcript molecule, facilitating haplotype phasing and detection of SNVs and indels. Allele-specific expression (ASE) in individual cells can reveal how genetic variation impacts specific cell states, lineages, or disease-associated subpopulations, which is very relevant in diseases with clonal mutations like cancer.

Post cell annotation, variants can be called using *ClairS* [137], *sniffles3* [138] or *longshot* [133], using combined data from clusters representing individual cell types. Variant calling for SCLRS can also be performed via inbuilt options of *FLAMES*, *SiCeLoRe*, and the standalone tool *LongSom*.

By integrating variant information with expression and splicing data, SCLR-seq can be used for analyses such as allele-specific expression mapping, isoform expression quantitative trait locus (eQTL) discovery, and functional interpretation of disease-associated variants in single cells, as described in Zhu *et al*. [139].

### Visualizing differential isoform usage

Visualization is critical for interpreting isoform-level changes across cell types or conditions. Several tools support this step in SCLR-seq workflows. *ScisorWiz* [109] is a tool specific to SCLR-seq for visualizing differential isoform expression data across multiple clusters. Other general-purpose visualization tools include: *ggsashimi* [127] for visualizing exon-level read coverage and splice junctions, *ggtranscript* [111] an R package for drawing isoform models and comparisons, and Integrative Genomics Viewer, which is widely used for viewing read alignments, including long reads, with support for custom annotations.

Together, these tools facilitate detailed examination of isoform architecture and support discovery of cell-type-specific or disease-associated splicing patterns.

## Application of single-cell long-read sequencing in human health research

A deeper understanding of molecular mechanisms at the transcriptome level can be facilitated by identifying splicing patterns at single-cell resolution. SCLR-seq is proving instrumental in studying diverse biological contexts, neurodevelopment, and immune cell diversity, and it is extremely useful in deciphering isoform expression patterns in cancer [66, 67, 76, 134, 140]. Dondi *et al*. sequenced clinical samples of ovarian cancer and found that mesothelial cells transition into cancer associated fibroblasts, partly via the TGF-beta (transforming growth factor)/miR-29/Collagen axis in omental metastases, by analyzing differential polyadenylation sites and differential isoform usage in collagen-encoding genes *COL1A2, COL3A1, COL5A2*, and *COL6A1* [66]. They also resolved gene fusion events that were misclassified in matched short-read data, further demonstrating the value of SCLR-seq in oncology and personalized medicine. Li *et al*. used SCLR-seq to explore the transcriptomic complexity of colorectal cancer, generating a comprehensive isoform atlas [134]. They found novel isoforms in tumor epithelial cells, many arising from complex combinations of splicing events. Some of these isoforms were unique to tumor cells and were experimentally validated. They showed that certain isoforms could generate neoepitopes with strong binding affinities to MHC molecules, suggesting their potential use in developing broadly applicable neoantigen-based cancer vaccines. Yang *et al*. sequenced single cells from cerebral organoids on both short- and long-read platforms and found that AS events of autistic brains are closer to the progenitor state rather than differentiated neurons, highlighting the importance of cell-type-specific splicing in autism [141].

In a recent study, Dondi *et al*. developed a method to call *de novo* somatic variants in ovarian cancer, facilitating the study of cancer evolution, clonal heterogeneity, and treatment resistance from SCLR-seq data [106]. Byrne *et al*. investigated the transcriptional complexity associated with genetic alterations in cancerous cells by associating differentially expressed isoforms to SNVs [68]. Similarly, Wedemeyer’s group leveraged SCLR-seq to uncover distinct isoform diversity and transcriptomic profiles in *PIK3CA*-mutant fibroblasts from capillary malformations, providing insights into the molecular consequences of low-frequency mosaic variants in PROS (PIK3CA-related overgrowth spectrum) disorders [142].

Beyond somatic variant discovery, SCLR-seq enables ASE analysis and haplotype phasing, allowing the dissection of how *cis*-regulatory variants affect transcript expression in individual cells. The ability to observe full-length, phased transcripts makes ASE analysis informative for studying allelic regulation, monoallelic expression, and splice site usage in heterogeneous cell populations. In a recent large-scale study, long-read sequencing of B cells from 67 individuals uncovered over 17 000 isoform eQTLs, more than 70% of which were missed by conventional gene-level analysis. Many isoform eQTLs were enriched at splice sites and regulatory histone marks (e.g. H3K36me3, H3K4me1), suggesting that they modulate expression via splicing and transcriptional regulation. Experimental validation using genome editing and minigene splicing assays further confirmed that some functional variants driving isoform expression are overlooked by existing splicing prediction tools [143].

A compelling application of SCLR-seq integrates with spatial transcriptomics to map isoform changes during human brain development. Traditional spatial transcriptomics, with resolutions around ~60 μm, often captures multiple cells per spot. Spl-ISO-seq, a newly developed spatial isoform sequencing method, improves resolution to ~10 μm approaching single-cell granularity to further resolve isoform expression. Spl-ISO-seq, when applied to the human visual cortex across pre- and postpubertal stages, revealed cortical layer and cell-type-specific regulation of AS and poly(A) site usage [144]. A novel application of SCLR-seq involves combining it with chromatin accessibility profiling to better understand how regulatory landscapes influence splicing. In the ScISOr-ATAC framework, lrRNA-seq and ATAC-seq are performed simultaneously on nuclei from frozen brain tissue, enabling joint analysis of chromatin accessibility and isoform usage in the same single cells. Hu *et al*. applied this technique to profile human and macaque brain cortices, including tissue from individuals with Alzheimer’s disease, to investigate how splicing and chromatin states of cells are regulated. For example, oligodendrocytes exhibit pronounced chromatin and splicing changes in Alzheimer’s disease, whereas astrocytes show chromatin dysregulation without major splicing effects [145].

## Discussion

Several SCLR-seq datasets have recently become available, reflecting the rapid growth and diversification of this field. Representative studies, including LR-Split-seq [78], ScISOr-Seq [67], the PacBio PBMC dataset (https://downloads.pacbcloud.com/public/dataset/Kinnex-single-cell-RNA/), and Nanopore-based single-cell transcriptome datasets [68, 146], have profiled complex tissues such as the human and mouse brain, liver, and various cancers. In addition, the recently developed LongBench resource [147] aimed at establishing a standardized reference for cross-platform performance assessment, provides a matched, multi-platform benchmark dataset spanning bulk, single-cell, and single-nucleus transcriptomics. It is from eight human lung cancer cell lines, including ONT (PCR-cDNA and direct RNA) and PacBio Kinnex data alongside Illumina short reads. These datasets employ distinct sequencing platforms, read depths, and library preparation strategies, offering complementary insights into isoform diversity and transcriptome complexity at single-cell resolution. Collectively, they provide a valuable foundation for benchmarking analytical methods and for exploring how lrRNA-seq can uncover cell-type–specific isoforms and aberrant splicing events relevant to human diseases.

SCLR-seq library preparation strategies have a substantial impact on the quality, resolution, and interpretability of data. Key choices in input material, barcoding method, and sequencing platform shape transcript recovery, isoform diversity, and downstream analysis. scRNA-seq captures cytoplasmic transcripts, including mature mRNAs, and is generally richer in isoform content, whereas snRNA-seq captures nuclear RNA, including unspliced or partially spliced transcripts, which can be advantageous for studying nascent transcription or nuclear-retained isoforms, but often underrepresents full-length mRNA.

Barcoding and reverse transcription platform affects both transcript coverage and barcode retention. Droplet-based platforms like 10× Genomics offer high-throughput and standardized barcoding but are limited by 3′ or 5′ capture bias, often losing full-length information and requiring specialized adaptations to support long-read sequencing. In contrast, plate-based full-length protocols such as SMART-seq3 offer more uniform transcript coverage across the entire mRNA and are better suited for long-read sequencing, albeit at lower throughput and higher cost per cell. The choice of long-read sequencing platform, PacBio versus ONT, also impacts data quality and read characteristics. PacBio HiFi reads offer higher per-base accuracy (≥99.9%), making them ideal for distinguishing similar isoforms or phasing allelic variants, though at lower throughput and higher cost. ONT provides higher throughput, portability, and flexible run configurations, but has higher error rates and greater variability in read length and quality. These trade-offs affect downstream isoform quantification, error correction needs, and feasibility for high-complexity samples.

Together these variables create a matrix of trade-offs between accuracy, throughput, cost, and isoform resolution. Ultimately, the choice of library preparation should be guided by the biological question, target transcript complexity, and available resources.

## Challenges and limitations of SCLR-seq

SCLR-seq faces a set of unique computational and technological challenges apart from the obvious limitations of sequencing long reads and sequencing in single cells. When compared to short-read next-generation sequencing (NGS) platforms, TGS platforms generally exhibit lower throughput and higher error rates. Although advances in error correction by ONT and increased throughput by PacBio have greatly improved the efficiency of SCLR-seq, these platforms are still often paired with NGS reads to increase confidence. Higher error rates complicate CB and UMI detection, leading to reduced accuracy in transcript quantification, thus requiring accompanying short-read data.

Single-cell sequencing data are inherently sparse and noisy due to multiple factors. Dropout events occur when gene abundance is too low to be reliably detected, appearing as zeros in the count matrix. The degree of sparsity reflects both true biological variation and methodological noise, which can hinder downstream analyses. In addition to batch effects, technical variability is amplified by amplification bias arising from low starting material. Collectively, these factors can produce incomplete or distorted expression profiles, reducing the reliability of predictions.

Applying long-read sequencing at the single-cell level presents challenges in reconstructing full-length isoforms from noisy, sparse, cell-specific data. Specialized computational tools are needed to address the unique characteristics of SCLR-seq. For example, algorithms must ensure accurate UMI detection to prevent UMI crowding, a phenomenon in which the edit distance between true UMIs decreases as gene expression increases, leading to ambiguous UMI assignment [108]. The use of UMIs helps identify PCR artifacts and reduce amplification bias, making accurate UMI detection essential. Zajac *et al*. compared technical characteristics by sequencing the same single-cell (10×) libraries on Illumina’s NovaSeq 6000 and PacBio’s Sequel IIe platforms, finding substantial overlap in the results from both [148]. However, genes with high counts in Illumina data were, on average, longer, whereas those with high counts in PacBio data tended to be shorter. In addition to this gene-length bias, Illumina reads mapping to intronic or intergenic regions were often discarded, while PacBio reads provided greater resolution in these regions [148].

Higher throughput can be achieved by sequencing single-cell transcriptomes with short reads rather than long reads at comparable cost. Beyond the expense of single-cell library preparation, research labs face high sequencing costs associated with TGS platforms. Consequently, SCLR-seq experiments remain costly, limiting their accessibility for studies targeting rare cell types or requiring deep sequencing to quantify low-abundance isoforms.

SCLR-seq workflows require significant computational resources for processing and storage. PacBio’s Revio sequencer can generate up to 360 Gb of data per day, and ONT’s PromethION 48 sequencer can generate a theoretical maximum yield of 14 Tb (when flow-cells at maximum capacity run together for 72 h) [149]. The need for file conversions and preprocessing for downstream analysis further increases the computational data burden, making efficient data storage and computational power critical for successful analysis. Analysis tools are often tailored to a specific experiment type and may not work on a wider range of customized barcodes and sequencing data, specifically, when the structure of the barcode, flanking sequences, and their locations differ from the usual specifications. Much of the available software is complex to set up and install, and its computational requirements in terms of time and memory can limit its practical use when processing the volumes of data now being generated. Moreover, the high costs of commercial kits and capital investment required for SCLR-seq limit the accessibility and flexibility of SCLR-seq. Furthermore, emerging tools have yet to be fully optimized for SCLR-seq analysis, resulting in gaps that limit effective downstream processing.

## Conclusions

SCLR-seq overcomes the limitations of conventional short-read single-cell approaches, transforming our ability to study AS at single-cell resolution. Recent advances in both experimental protocols and bioinformatics tools have addressed many initial technical challenges, enabling more accurate and scalable analyses. While short-read sequencing offers higher coverage at lower cost and facilitates barcode deconvolution to improve data reliability, many current workflows still rely on complementary short-read data to boost confidence in cell assignment and transcript quantification. As SCLR-seq technologies continue to mature and become more cost-effective, they may soon be used independently of short reads. Beyond transcriptome reconstruction, SCLR-seq can also integrate well into multi-modal single-cell frameworks where transcript isoform information can be combined with epigenomic, proteomic, or spatial data to provide a more comprehensive view of cellular identity and regulatory mechanisms. This multi-layered perspective will be valuable for dissecting complex diseases, developmental processes, and cell–cell interactions. SCLR-seq holds great promise for uncovering novel molecular insights and deepening our understanding of transcriptomic diversity in health and disease. Continued development of specialized computational pipelines will be critical to fully harness its potential in translational research and precision medicine.

**Key Points**

- Dysregulation of alternative splicing (AS), a major regulatory mechanism in gene expression, contributes to various human diseases, including cancer.
- This dysregulation has been understood by quantifying transcript isoforms through RNA-seq technologies such as single-cell short-read RNA-seq (scRNA-seq) and bulk lrRNA-seq.
- Single-cell long-read RNA sequencing (SCLR-seq) offers a better opportunity to capture isoform diversity than scRNA-seq or lrRNA-seq alone.
- This review summarizes recent advances in bioinformatics tools and pipelines for SCLR-seq data analysis and highlights how these developments have been used in human health research.

## Data Availability

There are no new data associated with this article.
